# X-linked Retinitis Pigmentosa in Japan: Clinical and Genetic Findings in Male Patients and Female Carriers

**DOI:** 10.3390/ijms20061518

**Published:** 2019-03-26

**Authors:** Kentaro Kurata, Katsuhiro Hosono, Takaaki Hayashi, Kei Mizobuchi, Satoshi Katagiri, Daisuke Miyamichi, Sachiko Nishina, Miho Sato, Noriyuki Azuma, Tadashi Nakano, Yoshihiro Hotta

**Affiliations:** 1 Department of Ophthalmology, Hamamatsu University School of Medicine, 1-20-1, Handayama, Higashi-ku, Hamamatsu, Shizuoka 431-3192, Japan; 41222975@hama-med.ac.jp (K.K.); hosono@hama-med.ac.jp (K.H.); dk0033@hama-med.ac.jp (D.M.); mihosato@hama-med.ac.jp (M.S.); 2 Department of Ophthalmology, The Jikei University School of Medicine, 3-19-18, Nishi-shimbashi, Minato-ku, Tokyo 105-8471, Japan; taka@jikei.ac.jp (T.H.); kei10151202@icloud.com (K.M.); ktgr_two_ai@icloud.com (S.K.); tnakano@jikei.ac.jp (T.N.); 3 Department of Ophthalmology and Laboratory for Visual Science, National Center for Child Health and Development, 2-10-1, Okura, Setagaya-ku, Tokyo 157-8535, Japan; nishina-s@ncchd.go.jp (S.N.); azuma-n@ncchd.go.jp (N.A.)

**Keywords:** retinitis pigmentosa, X-linked inheritance, *RPGR*, *RP2*, visual function, genetic findings

## Abstract

X-linked retinitis pigmentosa (XLRP) is a type of severe retinal dystrophy, and female carriers of XLRP demonstrate markedly variable clinical severity. In this study, we aimed to elucidate the clinical findings of male patients with and female carriers of XLRP in a Japanese cohort and demonstrate the genetic contribution. Twelve unrelated families (13 male patients, 15 female carriers) harboring pathogenic mutations in *RPGR* or *RP2* were included, and comprehensive ophthalmic examinations were performed. To identify potential pathogenic mutations, targeted next-generation sequencing was employed. Consequently, we identified 11 pathogenic mutations, of which five were novel. Six and five mutations were detected in *RPGR* and *RP2*, respectively. Only one mutation was detected in ORF15. Affected male patients with *RP2* mutations tended to have lower visual function than those with *RPGR* mutations. Female carriers demonstrated varying visual acuities and visual fields. Among the female carriers, 92% had electroretinographical abnormalities and 63% had a radial autofluorescent pattern, and the carriers who had higher myopia showed worse visual acuity and more severe retinal degeneration. Our results expand the knowledge of the clinical phenotypes of male patients with and female carriers of XLRP and suggest the possibility that *RP2* mutations are relatively highly prevalent in Japan.

## 1. Introduction

Retinitis pigmentosa (RP) is an inherited retinal disease that affects 1 in 3000 to 5000 individuals worldwide [1]. It involves progressive visual dysfunction, including night blindness, visual field constriction, and eventually central visual loss. Funduscopic findings show bone-spicule retinal pigmentations, chorioretinal atrophy, attenuated retinal vessels, and waxy optic disc pallor. Electroretinography (ERG) often reveals severely reduced or nondetectable response.

RP inheritance can be autosomal dominant (adRP), autosomal recessive (arRP), and X-linked recessive (XLRP). To date, mutations in over 80 genes have been associated with RP. XLRP is the most severe subtype of this disease and accounts for 10–20% of all RP cases [2]. Presently, mutations in three genes, *RP2* (OMIM312600), *OFD1* (OMIM300170), and *RPGR* (OMIM312610) have been identified as causative in XLRP. According to Caucasian cohorts, mutations in *RPGR* are found in approximately 30–80% of families with XLRP, whereas mutations in *RP2* are found in 10–20% [3,4,5,6]. In addition, mutations in *RPGR* or *RP2* account for 15% of isolated male patients with RP [7]. Male patients with XLRP generally show a more severe phenotype than female carriers. It has been reported that female carriers of XLRP show a wide spectrum of clinical features that can vary from asymptomatic to severe symptoms similar to those observed in male patients [7,8,9,10].

Although there have been many reports on the clinical findings or the genetic contribution in XLRP in various ethnicities [11,12,13], only limited data from the Japanese population are available [14,15]. The prevalence of XLRP in Japan is considered to be very low; it is reported at 1.6% of all RP cases [16,17]. Currently, gene therapy in *RPGR* or *RP2* animal models is proceeding [18,19,20]. Therapeutic efficacy would be more accurate if the disease phenotype was elucidated. Therefore, it is important to describe the clinical findings of Japanese patients with XLRP. The purpose of this study was to assess the clinical findings in male affected with XLRP and female carriers, as well as the genetic contribution of XLRP in the Japanese population.

## 2. Results

### 2.1. Potentially Pathogenic Mutations in the Subjects

**Figure 1:**
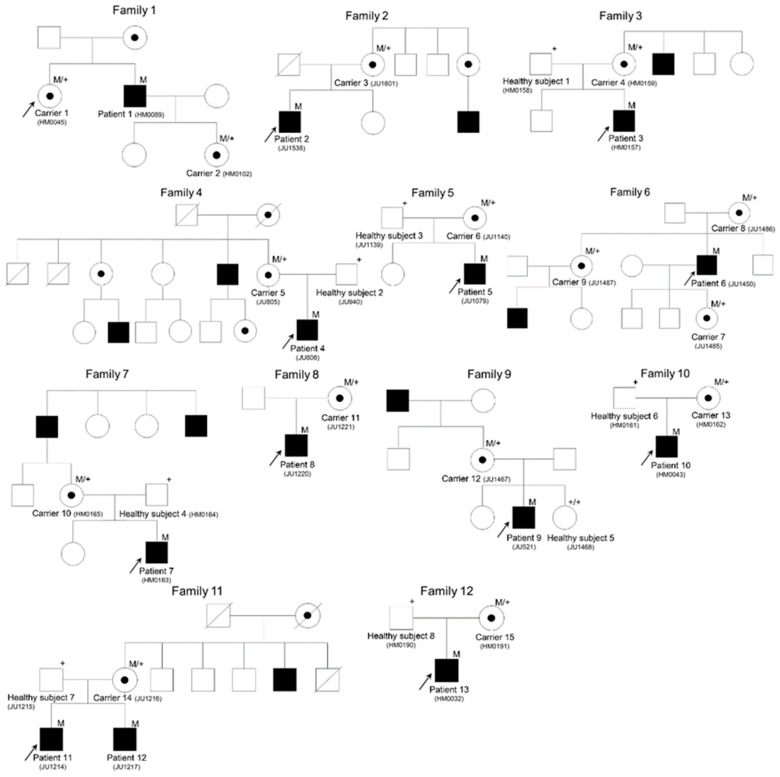
The pedigrees of the families in this study. Closed and open symbols represent affected and unaffected individuals, respectively. Dotted circles represent female carriers. Arrows indicate probands. The slash indicates a deceased person. M refers to the mutant allele and + to the normal allele.

In total, 13 affected male subjects and 15 carrier female subjects from 12 families were recruited. Of the 12 families, seven carried the typical X-linked mode of inheritance and the other five were isolated cases (Figure 1).

We carried out targeted next-generation sequencing (TS) to identify pathogenic mutations in the subjects. The obtained sequence data were analyzed using our bioinformatics pipeline [21]. Consequently, in the 12 analyzed families, we identified 11 potential pathogenic mutations, of which five were novel. All potential pathogenic mutations identified in this study were confirmed by Sanger sequencing.

We found six mutations in *RPGR*, which consisted of two nonsense, one frameshift, one splice site, and two missense mutations in seven families (Families 1–7) (Table 1). Two families (Families 5 and 6) had a c.1234C>T mutation. Family 7 had a c.2997_2998del mutation in the *RPGR* exon of the ORF15 region. Additionally, we found five mutations in *RP2*, which consisted of two nonsense, one frameshift, one splice site, and one missense mutation in five families (Families 8–12) (Table 1). Our TS data from the 12 analyzed families revealed no causative genes other than *RPGR* or *RP2*. These data suggest that the causative gene in these families was either *RPGR* or *RP2*. All variants co-segregated in affected male and carrier female subjects. None of the potential pathogenic mutations were found in healthy relatives of these families. The pathogenicity of the novel missense mutations was supported by in silico prediction analyses results (Table S1).

### 2.2. Clinical Findings of Affected Male Subjects

**Figure 2:**
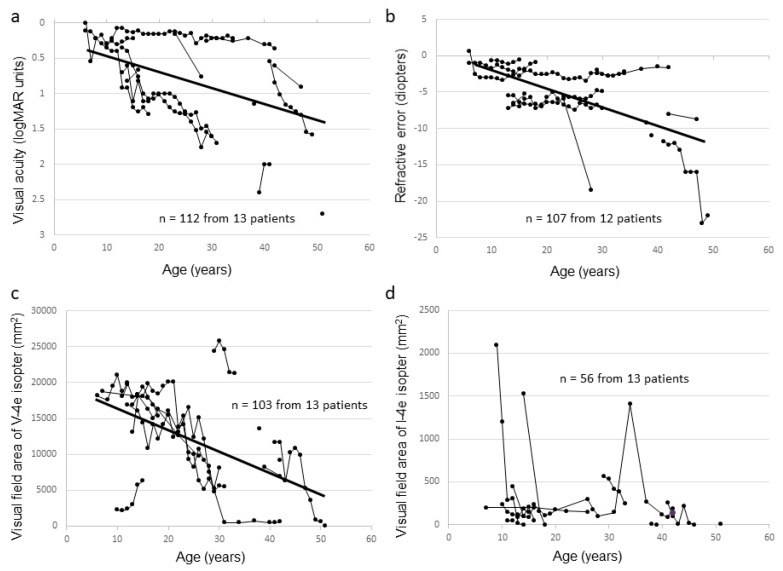
Correlation between visual function and age among affected male subjects in this study. The graphs show visual acuity values expressed in logMAR units (**a**), refractive error (**b**), visual field extent with the V-4e isopter (**c**), and visual field extent with the I-4e isopter (**d**) versus age. The data from the same subject are indicated using bars connecting the points.

Seven affected male subjects from seven families had mutations in *RPGR*, whereas six affected male subjects from five families had mutations in *RP2*. The clinical data of affected male subjects are summarized in Table 2 (the clinical information of each subject is shown in Table S2). The median age was 34 years (range, 15–54 years). All affected male subjects had night blindness or visual loss, with the ages at onset ranging from early childhood to the age of 11 years (median, six years). The best corrected visual acuity (BCVA) at first visit ranged from 0 logMAR units to light perception, and BCVA deterioration correlated with increasing age (r = 0.41, *p* < 0.001) (Figure 2a). The BCVA was relatively preserved up to the 1st decade and started to worsen early in the 2nd decade reaching light perception at approximately 50 years of age. Refractive error became more negative with increasing age (r = −0.51, *p* < 0.001) (Figure 2b). The visual field area evaluated with the V-4e isopter became narrower with increasing age (r = −0.51, *p* < 0.001), and no effective residual visual field was observed in the 5th decade (Figure 2c,d). Optic coherence tomography (OCT) images showed a marked decrease in retinal thickness in all affected male subjects, and cystoid macular edema (CME) was not seen. All affected male subjects had a non-recordable response on ERG except for one who had a residual photopic response.

### 2.3. Clinical Findings of Female Carriers

**Figure 3:**
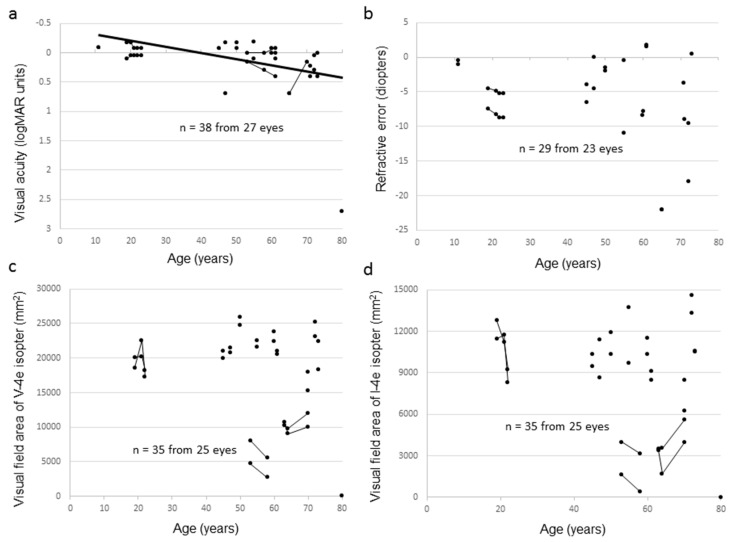
Correlation between visual function and age among carrier female subjects in this study. The graphs show visual acuity values expressed in logMAR units (**a**), refractive error (**b**), visual field extent with the V-4e isopter (**c**), and visual field extent with the I-4e isopter (**d**) versus age. The data from the same subject are indicated using bars connecting the points.

**Figure 4:**
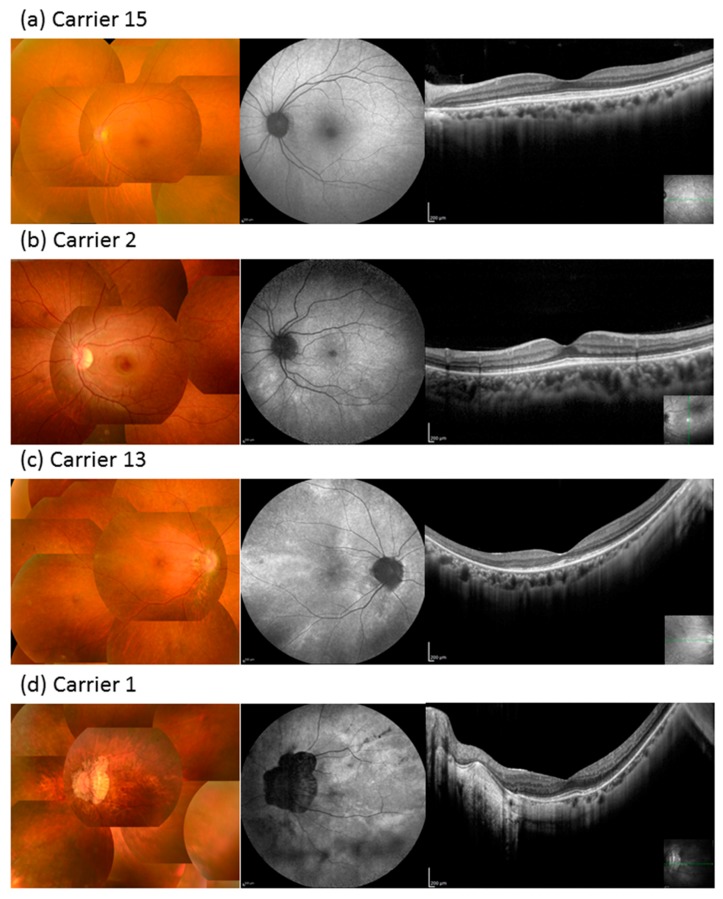
Color fundus photography, fundus autofluorescence (FAF), and optic coherence tomography (OCT) of carrier female subjects in this study. (**a**) Fundus findings of Carrier 15. Color fundus photography revealed no abnormalities (grade 0). FAF revealed a somewhat radial pattern. No obvious abnormalities were detected on OCT. (**b**) Fundus findings of Carrier 2. Tapetal reflex was detected temporal to the macula (grade 1). FAF revealed a radial pattern and there were no obvious abnormalities on OCT. (**c**) Fundus findings of Carrier 13. Pigmentary change was detected in the temporal peripheral retina (grade 2). FAF showed a radial autofluorescent pattern. The ellipsoid zone was extinguished in the temporal retina on OCT. (**d**) Fundus findings of Carrier 1. Diffuse retinal atrophy including pigmentary disturbance was observed (grade 3). FAF showed a radial autofluorescent pattern and hypoautofluorescence consistent with retinal pigment epithelium atrophy. OCT showed severe atrophy of the outer retina and choroid. Scar bar = 200 µm.

Ten carrier female subjects from seven families had mutations in *RPGR*, whereas five carrier female subjects from five families had mutations in *RP2*. The clinical data of carrier female subjects are summarized in Table 3 (the clinical information of each subject is shown in Table S3). The median age of the carrier female subjects was 59.5 years (range, 11–80 years). Night blindness and photophobia were observed in five and two carrier female subjects, respectively, and four carrier female subjects were asymptomatic. Carrier 3 received surgery in her left eye due to retinal detachment at the age of 40 years and, thus, her left eye was excluded from the analysis. The BCVA varied considerably and ranged from −0.18 logMAR units to light perception. Nine carrier female subjects (64.3%) had decimal BCVA of 1.0 or better in at least one eye and only one had legal blindness. BCVA deterioration correlated with increasing age (r = 0.59, *p* < 0.001) (Figure 3a). Almost all carrier female subjects without cataract extraction showed moderate to high myopia from –22.0 to +1.75 diopters (median, −3.88 diopters), which was independent of age (r = −0.19, *p* = 0.30) (Figure 3b). Anisometropia, denoted by > 1.0 diopter of spherical equivalents between the right and left eye, was observed in six carrier female subjects. The extent of the visual field varied (Figure 3c,d), and 56% of eyes had abnormal visual fields such as concentric constriction or scotoma. Most carrier female subjects had normal OCT findings; however, there were abnormalities in a small number of subjects, such as disruption of the ellipsoid zone or thinning of the outer retinal layer, and CME was not seen in any carrier female subject. The full-field ERG responses varied between non-detectable and within the normal range. Fundus autofluorescence (FAF) was performed on 27 eyes of 14 carriers. Of these, 17 eyes had a radial autofluorescent pattern (Figure 4).

### 2.4. Phenotype-Genotype Correlation

**Figure 5:**
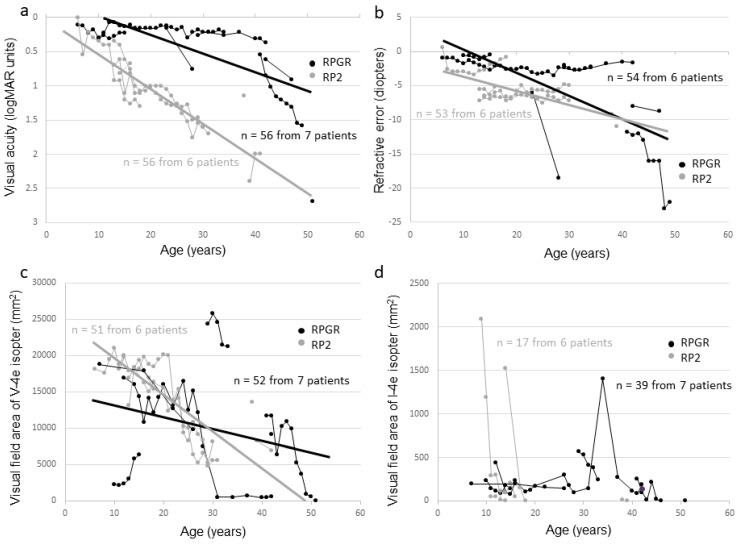
Correlation between visual function and age among affected male subjects with *RPGR* or *RP2* mutation. Black dots and lines indicate the data of affected male subjects with *RPGR* mutation and gray dots and lines indicate the data of affected male subjects with *RP2* mutation. The graphs show visual acuity values expressed in logMAR units (**a**), refractive error (**b**), visual field extent with the V-4e isopter (**c**), and visual field extent with the I-4e isopter (**d**) versus age. The data from the same subject are indicated using bars connecting the points.

Figure 5 compares various ocular functions by age for affected male subjects with *RP2* or *RPGR* mutations. For both affected male subjects with *RPGR* or *RP2* mutations, BCVA deterioration correlated with increasing age (r = 0.65, *p* < 0.001, and r = 0.90, *p* < 0.001, respectively). The difference between the patients with *RPGR* and the patients with *RP2* mutations is that affected male subjects with *RP2* mutations tended to have lower visual acuities across all ages (Figure 5a). The refractive error became more negative with increasing age in affected male subjects without obvious difference between those with *RPGR* and those with *RP2* mutations (r = −0.75, *p* < 0.001 and r = −0.50, *p* < 0.001, respectively) (Figure 5b). The visual field area evaluated with the V-4e isopter of the affected male subjects with *RPGR* or *RP2* mutations revealed that deterioration correlated with increasing age (r = −0.34, *p* = 0.018 and r = −0.79, *p* < 0.001, respectively) (Figure 5c). The visual field areas of patients with *RP2* mutations tended to have a faster rate of deterioration than those of patients with *RPGR* mutations (Figure 5c,d).

The visual function of the female subjects carrying either *RPGR* or *RP2* mutation was variable and less predictable with age. There were no obvious differences between *RPGR* and *RP2* carriers with respect to BCVA, visual field area, but myopia was significantly more severe in female carriers with *RPGR* mutation (Table 3).

### 2.5. Severity Among the Female Carriers

**Figure 6:**
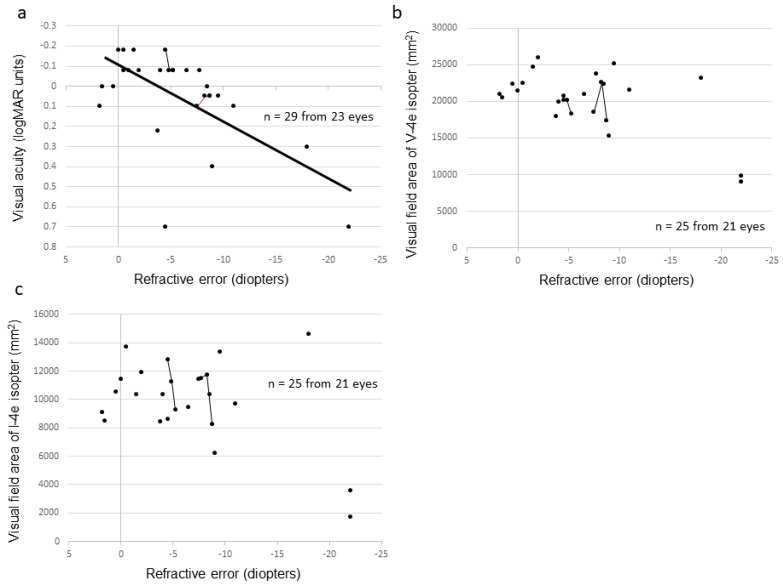
Correlation between refractive error and visual function among carrier female subjects. The graphs show visual acuity values expressed in logMAR units (**a**), visual field extent with the V-4e isopter (**b**), and visual field extent with the I-4e isopter (**c**) versus refractive error. The data from the same subject are indicated using bars connecting the points.

Figure 6 shows the correlation between refractive error and visual function in carrier female subjects. The carriers who had higher myopia showed worse visual acuity (*p* = 0.002) (Figure 6a). Although the carriers who had higher myopia tended to show a narrower visual field area, there was no statistically significant difference (Figure 6b,c). As for the fundus appearance, 15%, 33%, 19%, and 33% of eyes had grade 0, 1, 2, and 3, respectively (Figure 4) [25]. The mean age, BCVA, refractive error, and visual field area evaluated with the V-4e isopter were related to the grade of fundus appearance (*p* = 0.001, *p* = 0.005, *p* = 0.001, and *p* = 0.046, respectively, Jonckheere–Terpstra (J–T) test, Table 4), while the visual field area evaluated with the I-4e isopter was not related to the grade of fundus appearance (*p* = 0.062).

**Figure 7:**
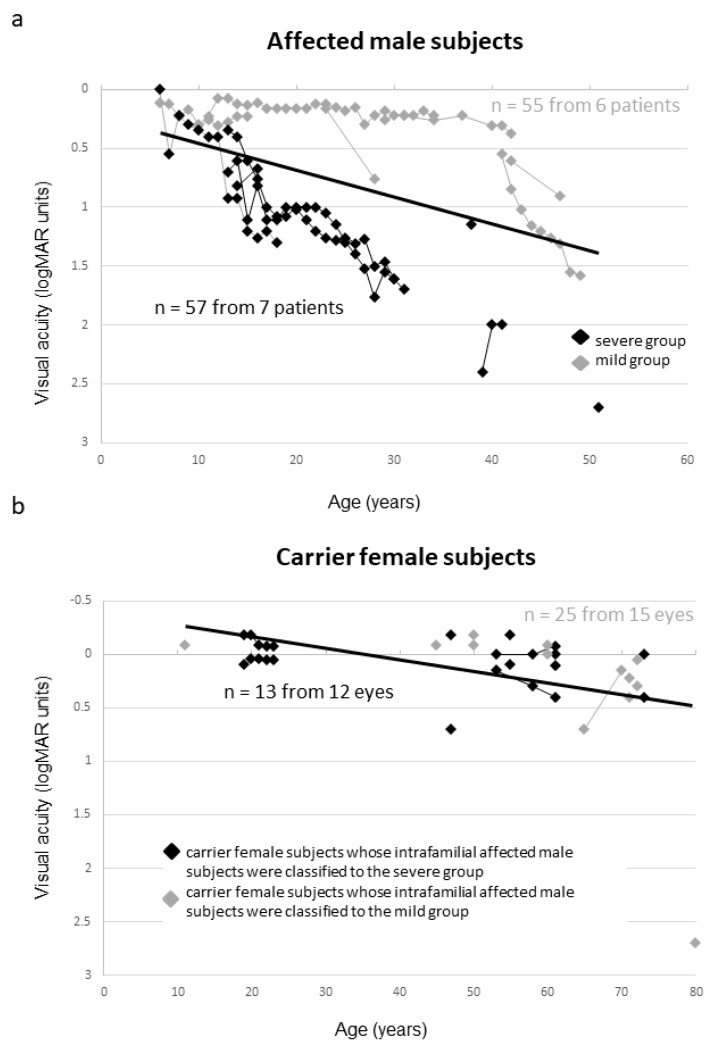
Association of the severity between affected male and carrier female subjects. The data from the same subject are indicated using bars connecting the points. (**a**) Black and gray diamonds indicate the severe and mild phenotypic groups, respectively. Affected male subjects were divided into two groups according to their phenotypic severity. Visual acuity above the regression line was defined as mild (mild group). Conversely, visual acuity below the regression line was defined as severe (severe group). (**b**) The data of carrier female subjects whose intrafamilial affected male subjects were classified into the severe group are indicated with black diamonds. Conversely, the data of carrier female subjects whose intrafamilial affected male subjects were classified into the mild group are indicated by gray diamonds.

We investigated the association of severity between affected male and carrier female subjects. As shown in Figure 7a, affected male subjects were divided into two groups according to their phenotypic severity (see the Figure 7 legend). Figure 7b shows the carrier female subjects according to their intrafamilial affected male severities. There was no obvious association of phenotypic severity between affected male and carrier female subjects.

## 3. Discussion

The present study described mutational and clinical findings of Japanese patients with XLRP caused by *RPGR* or *RP2* mutation. In the present study, most of the affected male subjects were aware of visual disturbance during the 1st decade of life, and the visual acuity and visual fields of these subjects declined with increasing age and tended to be worse in the patients with *RP2* mutations. These findings were consistent with those of a previous study [11].

The carrier female subjects in our study revealed variability in phenotypic severity that ranged from no visual changes to severe visual loss. The visual field of the female carriers was relatively stable with increasing age. In contrast, the visual acuity of the female carriers worsened with increasing age although at a slower rate than that of the affected male subjects. There were no differences in phenotypic severity between *RPGR* and *RP2* carriers. Moreover, there was no obvious association in phenotypic severity between affected male and carrier female subjects. Leber congenital amaurosis (LCA) caused by *RPGR* or *RP2* mutation, which is one of the most severe forms of inherited retinal dystrophy, was reported in a Chinese cohort [26,27]. Although not shown in the reports, it would be interesting to know the severity of the phenotype of female carriers of LCA-causing mutations.

The stability of visual function in the female carriers correlated well with fundus appearance: the more severe the retinal degeneration, the worse the visual acuity. This finding was consistent with those of previous studies [25,28]. In contrast, retinal degeneration was more apparent in carriers with older age, which was not consistent with the findings of a previous study [25]. Grover et al. reported that the grade of fundus findings did not change over an extended follow-up period [25]. In the current study, retinal degeneration was also more apparent in carriers with high myopia. This signifies that retinal degeneration may be influenced by myopia. In addition, carriers with high myopia showed more deteriorated visual acuity. Visual acuity in our carrier female subjects was worse than that previously reported; however, in that study, the subjects had milder myopia [29]. Although skewed X-activation, age, environmental factors, and genetic modifiers are classically considered the reason for the variability of phenotypic severity in female carriers [3,10], our findings suggest that myopic level may also play a role in phenotypic severity. Anisometropia was observed in 55% of the female carriers, which is higher than that reported for a healthy Japanese cohort (15.1%) [30]. Anisometropia often causes amblyopia; therefore, suitable refractive correction from an early stage is needed.

In this study, 92% and 63% of female carriers had abnormal ERG and radial autofluorescence on FAF, respectively, which is in line with previously reported findings [10,31]. Although some studies have reported radial autofluorescence on FAF in female carriers of *RPGR* mutation [32,33], we showed that female carriers of *RP2* mutation also had a radial autofluorescence pattern on FAF. The reason why female carriers of *RPGR* mutation have a radial autofluorescence pattern was speculated by Wegscheider et al. [32]: X-chromosome inactivation and central-to-peripheral migration of stem cells. This speculation may apply to female carriers of *RP2* mutation. Although ERG or several fundus images may reportedly identify the XLRP carriers effectively [34,35], FAF is also effective given its low invasiveness and specific autofluorescent pattern. However, whether a woman is a carrier or not is a critical issue for marriage or family planning; therefore, not only clinical but also genetic testing is needed to accurately identify female carriers after considering the ethical implications.

According to previous studies in several ethnic groups, mutations in *RPGR* are found in approximately 30–80% of families with XLRP, whereas mutations in *RP2* in 10–20% [3,4,5,6]. In addition, approximately 60% of disease-causing mutations in *RPGR* are found in ORF15 [4]. In this study, we found *RPGR* mutations in seven families, and of the *RPGR* mutations, only one was located in ORF15. Surprisingly, *RP2* mutations were detected in five families, which signifies higher prevalence than that in other ethnic groups. Of our 12 families, five had sporadic traits. In Japan, detailed family history cannot be often easily obtained because nuclear families are relatively common. Therefore, it is speculated that the isolated cases were in fact fewer.

A limitation of this study was the relatively small sample. However, subjects in this study were restricted to affected men and carrier women whose mutation in *RPGR* or *RP2* was detected via next-generation sequencing (NGS); hence, the clinical findings for XLRP in this study are considered to be valuable. Although it may be difficult to collect more samples in Japan where the prevalence of XLRP is relatively rare, a large sample is needed to examine the genotype-phenotype correlation or mutational spectrum more accurately. In addition, this study is not part of a cohort of patients with RP and, thus, we were unable to determine the frequency of XLRP in the population.

## 4. Materials and Methods

### 4.1. Ethics Statement

This study was approved by the Institutional Review Board for Human Genetic and Genome Research at the Hamamatsu University School of Medicine (permit no. 14-040), The Jikei University School of Medicine (permit no. 24-232 6997), and the National Centre for Child Health and Development (permit no. 686). All study procedures adhered to the tenets of the Declaration of Helsinki. Written informed consent was obtained from each subject or guardian before any study procedure or examination was performed.

### 4.2. Patient Recruitment

Japanese patients with RP suspected to have apparent X-linked recessive inheritance were recruited in the current study. In addition, we included isolated cases suspected of having XLRP mutations on the basis of clinical factors such as showing visual disorder in the first decade of life and rapid visual deterioration. An X-linked inheritance was strongly suggested in the case that there were at least two affected male patients in a family in the absence of comparably affected female relatives and no male-to-male transmission. Among these, affected male subjects and female carriers in whom mutation in *RPGR* or *RP2* was detected were finally enrolled. The criteria for diagnosing RP were based on night blindness, progressive visual loss, diminished responses on ERG without cone-rod dystrophy pattern, fundus abnormalities, such as bone-spicule pigmentation, attenuated retinal vessels, and waxy optic disc pallor.

### 4.3. Clinical Examination

Affected male subjects and female carriers were examined at Hamamatsu University School of Medicine and The Jikei University School of Medicine and underwent ophthalmic examinations including BCVA, refraction measurement, kinetic visual fields on the Goldmann perimeter, and full-field ERG. The values of these data for the right and left eyes were averaged in affected male subjects. Conversely, because XLRP female carriers show discordance between their right and left eyes [36], the data of both the right and left eyes were analyzed separately in these subjects. In addition, slit lamp biomicroscopy, ophthalmoscopy after pupillary dilation, fundus photography, FAF (Heidelberg Engineering, Heidelberg, Germany), and OCT (Carl Zeiss Meditec AG, Dublin, CA, USA and Heidelberg Engineering) were performed. Information on family history was obtained through interviews with subjects or their family members.

The BCVA was measured as decimal visual acuity using a Landolt C chart and converted to logMAR units for analysis. Visual acuity values for counting fingers vision, hand motion vision, and light perception vision were extrapolated to logMAR 2.0, 2.4, and 2.7, respectively [37]. Goldmann visual fields were captured using a digital scanner and areas of the V-4e and I-4e isopters were quantified using ImageJ [38]. The refractive error was recorded as spherical equivalent and was analyzed for subjects that had not undergone cataract surgery. The BCVA, visual field, and refractive error were examined on multiple occasions separated by at least 1 year for all affected male subjects except for Patient 12 and some carrier female subjects (Carriers 1, 2, and 5), and BCVA versus age, visual field areas versus age, and refractive error versus age were plotted and analyzed for the phenotype-genotype correlation. In addition, we graded the fundus appearance of female carriers, using the criteria suggested by Grover et al. [25]: normal-looking fundus (grade 0), tapetal-like retinal reflex only, without any peripheral pigmentary retinal changes (grade 1); regional peripheral pigmentary changes involving a quadrant or hemisphere, with or without a tapetal-like retinal reflex (grade 2); or three or more quadrants of diffuse pigmentary disturbances, including bone spicule-like pigment, hypopigmentation, and atrophic changes of the retinal pigment epithelium and choroid (grade 3).

### 4.4. Statistical Analysis

Statistical analyses were performed using SPSS version 25 (SPSS Japan Inc., Tokyo, Japan). Spearman’s rank correlation coefficient was used for the simple correlation tests. The J–T test was used to determine statistically significant differences between four groups of fundus grade with various visual function parameters. *p* values < 0.05 were considered statistically significant.

### 4.5. TS and Bioinformatics Analysis

A custom target enrichment library was designed to capture the 111 genes known to be associated with inherited eye diseases, as reported in RetNet or the Human Gene Mutation Database at the time of the system design (Table S4) [39,40]. The integrity of the TS approach used in this study has been evaluated previously [41]. The panel was designed, the library prepared, and target-capture sequencing was performed as previously described [42]. The sequence reads were mapped to the human reference genome sequence (GRCh37/hg19) using the Burrows-Wheeler Aligner software v. 0.7.15. The ANNOVAR software v 2016Feb01 was used to annotate single nucleotide variants and insertion-deletion polymorphisms. We focused on nonsynonymous variants and splice site variants, which are within 5 bp of the exon-intron boundaries (±5 bp), and excluded synonymous and non-coding exonic variants from the analysis. Details of the bioinformatics analysis and assessment of identified variants have been described previously [21].

### 4.6. Sanger Sequencing

The *RPGR* exon ORF15 is a mutational hotspot for X-linked RP; however, it contains repetitive sequences that cannot be efficiently captured or enriched by conventional targeted capture NGS [43]. Thus, genomic *RPGR* fragments encompassing the ORF15 region were amplified and analyzed by Sanger sequencing for the subjects for whom no potentially pathogenic variant(s) were identified by our TS approach [21].

Potential pathogenic mutations detected using the TS approach were validated by performing Sanger sequencing as per the standard protocol [43]. The refseq (accession number) references using in this study were NM_006915.3 (*RP2*), NM_000328.2/NM_001034853.1 (*RPGR*), and NM_001034853.1 (*RPGR-ORF15*). All utilized primers are available upon request. Sanger sequencing segregation analyses were performed on DNA from family members to investigate the co-segregation of potentially pathogenic mutations.

## 5. Conclusions

In summary, here, we studied the largest, to our knowledge, Japanese XLRP cohort. Affected male subjects with *RP2* mutations had a more severe phenotype than those with *RPGR* mutations. Female carriers in this study showed a wide spectrum of clinical features and their myopic level was associated with the degree of fundus degeneration and visual acuity. Although a study with a larger sample is needed, our results indicate that there may be a higher prevalence of *RP2* mutations in Japan compared to other regions. This study provided a comprehensive phenotyping and mutational spectrum of male patients with and female carriers of XLRP caused by *RPGR* or *RP2* mutations in the Japanese population.

## Figures and Tables

**Table 1:** Mutations found in *RPGR* or *RP2* in subjects in the present study.

Family Number	Subject Number	Gene	Accession Number	Mutation	Protein Change	Zygosity	Reference
1	Patient 1 Carrier 1	*RPGR*	NM_001034853.1 NM_000328.2	c.469+1G>T c.469+1G>T	p.? p.?	Hemi Hetero	[16]
	Carrier 2			c.469+1G>T	p.?	Hetero	
2	Patient 2	*RPGR*		c.492G>T	p.(W164C)	Hemi	Novel
	Carrier 3			c.492G>T	p.(W164C)	Hetero	
3	Patient 3	*RPGR*		c.830G>T	p.(G277V)	Hemi	Novel
	Healthy subject 1			WT	―	―	
	Carrier 4			c.830G>T	p.(G277V)	Hetero	
4	Patient 4	*RPGR*		c.1077T>A	p.(C359 *)	Hemi	Novel
	Healthy subject 2			WT	―	―	
	Carrier 5			c.1077T>A	p.(C359 *)	Hetero	
5	Patient 5	*RPGR*		c.1234C>T	p.(R412 *)	Hemi	[2]
	Healthy subject 3			WT	―	―	
	Carrier 6			c.1234C>T	p.(R412 *)	Hetero	
6	Patient 6	*RPGR*		c.1234C>T	p.(R412 *)	Hemi	[2]
	Carrier 7			c.1234C>T	p.(R412 *)	Hetero	
	Carrier 8			c.1234C>T	p.(R412 *)	Hetero	
	Carrier 9			c.1234C>T	p.(R412 *)	Hetero	
7	Patient 7	*RPGR (ORF15)*	NM_001034853.1	c.2997_2998del	p.(E1000Gfs *78)	Hemi	[2]
	Healthy subject 4			WT	―	―	
	Carrier 10			c.2997_2998del	p.(E1000Gfs *78)	Hetero	
8	Patient 8	*RP2*	NM_006915.3	c.102+1G>A	p.?	Hemi	[22]
	Carrier 11			c.102+1G>A	p.?	Hetero	
9	Patient 9	*RP2*		c.217del	p.(Y73Ifs *18)	Hemi	Novel
	Carrier 12			c.217del	p.(Y73Ifs *18)	Hetero	
	Healthy subject 5			WT	―	―	
10	Patient 10	*RP2*		c.358C>T	p.(R120 *)	Hemi	[23]
	Healthy subject 6			WT	―	―	
	Carrier 13			c.358C>T	p.(R120 *)	Hetero	
11	Patient 11	*RP2*		c.413A>G	p.(E138G)	Hemi	[24]
	Healthy subject 7			WT	―	―	
	Carrier 14			c.413A>G	p.(E138G)	Hetero	
	Patient 12			c.413A>G	p.(E138G)	Hemi	
12	Patient 13	*RP2*		c.685C>T	p.(Q229 *)	Hemi	Novel
	Healthy subject 8			WT	―	―	
	Carrier 15			c.685C>T	p.(Q229 *)	Hetero	

*: translation termination (stop) codon.

**Table 2:** Summary of the clinical characteristics of affected male subjects in the present study.

	All	Mutations in *RPGR*	Mutations in *RP2*	*p* Value
Number of subjects	13	7	6	
Median age (range)	34 (15–54)	42 (15–54)	30 (17–42)	0.19

Median onset age (range)	6 (1–11)	6 (1–9)	7.5 (3–11)	0.37

Median refractive error (range), diopter	−5.8 (−24.0–−0.50)	−5.5 (−24.0–−0.50)	−5.8 (−10.0–+1.00)	0.86

Median decimal BCVA (range)	0.08 (LP–0.7)	0.2 (LP–0.7)	0.03 (CF–0.3)	0.15

Median visual field area with V-4e (range), mm^2^	8089 (0–21,271)	6302 (0–21,271)	10814 (5528–20,048)	0.25

Median visual field area with I-4e (range), mm^2^	50 (0–244)	142 (0–244)	3 (0–52)	0.08


BCVA, best corrected visual acuity; LP, light perception; CF, counting fingers. Unpaired t test and Mann–Whitney U test were used for statistical analyses.

**Table 3:** Summary of the clinical characteristics of carrier female subjects in the present study.

	All	Mutations in *RPGR*	Mutations in *RP2*	*p* Value
Number of subjects	15	10	5	
Median age (range)	59.5 (11–80)	59.5 (11–80)	58 (47–73)	0.59

Visual disturbance, n, (%)	9/13 (69%)	7/9 (78%)	2/4 (50%)	0.66
Median refractive error (range), diopter	−3.88 (−22.0–+1.75)	−5.88 (−22.0–−0.50)	−0.25 (−11.0–+1.75)	0.02 *

Median decimal BCVA (range)	1.0 (LP–1.5)	1.0 (LP–1.5)	0.9 (0.2–1.5)	0.87

Median visual field area with V-4e (range), mm^2^	20,774 (38.1–25,947)	18,247 (38.1–25,947)	21,253 (18,339–22,501)	0.32

Median visual field area with I-4e (range), mm^2^	9448 (0–14,629)	9256 (0–14,629)	10,113 (8464–13,711)	0.22

Fundus grade, eyes, (%)				
Grade 0	4/27 (15%)	2/19 (11%)	2/8 (25%)	0.58
Grade 1	10/27 (37%)	6/19 (31%)	4/8 (50%)	0.70
Grade 2	4/27 (15%)	2/19 (11%)	2/8 (25%)	0.58
Grade 3	9/27 (33%)	9/19 (47%)	0/8 (0%)	0.16
Radial AF, eyes, (%)	17/27 (63%)	9/19 (47%)	8/8 (100%)	0.34
3.0 DA ERG patterns, eye, (%)				
No abnormalities	2/25 (8%)	0/17 (0%)	2/8 (25%)	0.13
Subnormal	9/25 (36%)	7/17 (42%)	2/8 (25%)	0.69
Reduced	9/25 (36%)	5/17 (29%)	4/8 (50%)	0.69
Non-detectable	5/25 (20%)	5/17 (29%)	0/8 (0%)	0.29

BCVA, best corrected visual acuity; LP, light perception; AF, autofluorescence; DA, dark adapted; ERG, electroretinography. Unpaired t test, Mann–Whitney U test, and Fisher’s exact test were used for statistical analyses. *: significant at *p* < 0.05.

**Table 4:** Relation of fundus grade and visual function in female carriers.

	Grade 0	Grade 1	Grade 2	Grade 3	J-T Test
Age (years)	53.0	35.7	62.6	67.6	*p* = 0.001 **
BCVA (logMAR unit)	−0.015	−0.079	0.20	0.47	*p* = 0.005 **
Refractive error (diopters)	−1.81	−3.29	−5.06	−14.04	*p* = 0.001 **
Visual field area (mm^2^)					
V-4e isopter	20,619	22,716	21,540	12,439	*p* = 0.046 *
I-4e isopter	9343	11,443	10,312	6198	*p* = 0.062

J-T, Jonckheere–Terpstra; BCVA, best corrected visual acuity. *: significant at *p* < 0.05, **: significant at *p* < 0.01.

## Supplementary Materials

Supplementary materials can be found at https://www.mdpi.com/1422-0067/20/6/1518/s1.
